# Assessment of health literacy in a French emergency department

**DOI:** 10.1186/s12913-024-11003-1

**Published:** 2024-04-22

**Authors:** Pauline Marie, Nicolas Romain-Scelle, Veronique Potinet, Anne Marie Schott, Marion Douplat

**Affiliations:** 1 Hospices Civils of Lyon, Hôpital Lyon Sud, Emergency department, Université Claude Bernard Lyon 1, 69495 Pierre-Bénite, France; 2 CNRS, Laboratoire de Biométrie et Biologie Évolutive UMR, 5558 Villeurbanne, France; 3 Université Claude Bernard Lyon 1, Research on Healthcare Performance (RESHAPE), INSERM, U1290 Lyon, France; 4 UMR ADéS 7268, Aix-Marseille Université/ EFS / CNRS, Espace éthique méditerranéen, Marseille, France

**Keywords:** Health literacy, Emergencies, Public health, Health status

## Abstract

**Background:**

Health literacy (HL) has become a subject of major interest in public health worldwide. It is known to be linked to self-efficacy in care use and to global health status, and a non-negligible frequency of problematic or inadequate levels of HL in populations worldwide is reported. As this has yet to be evaluated in France, the present study aimed to evaluate the HL level of patients in a French emergency department (ED).

**Methods:**

We conducted a descriptive, cross-sectional observational, single center study in the ED of the Lyon Sud hospital (Hospices civils de Lyon, Lyon, France). The primary endpoint was the HL level of the patients determined according to the score obtained using the 16-item European Health Literacy Survey Questionnaire. The secondary endpoint was the identification of sociodemographic factors associated with the HL level.

**Results:**

A total of 189 patients were included for analysis. 10% (95% CI [3%; 17%]) of the patients had an inadequate HL, 38% (95% CI [31%; 45%]) had a problematic HL, and 53% (95% CI [46%; 61%] had an adequate HL. In multivariate analysis, age and perceived health status were independent predictors of the HL level; OR =0.82 (95% CI [0.69; 0.97]; *p*=0.026) for a 10-year increase in age, and OR =1.84 (95% CI [1.22; 2.82]; *p*=0.004]).

**Conclusions:**

The HL level of the patients in the ED studied herein was similar to that found in the population of France and other European countries and was influenced by age and perceived health status, which are both associated with care needs. It may be therefore interesting to explore in future studies how taking into consideration HL in the general population may lead to a better self-efficacy in care and optimize the use of the healthcare system.

## Introduction

Health literacy (HL) has become a subject of major interest in public health worldwide [1]. According to Sørensen *et al.* (2012), HL is described as “people's knowledge, motivation, and competencies to access, understand, appraise, and apply health information to make judgments and take decisions in everyday life concerning healthcare, disease prevention, and health promotion to maintain or improve quality of life during the life course” [2]. Thus, HL is a concept of importance in all healthcare fields including prevention and promotion of health. HL is known to be linked to self-efficacy in care use and to global health status [3]. In addition a low HL is associated with negative health outcomes, higher morbidity of chronic diseases, and a lower use of preventive care [4], which leads to higher medical costs, a greater number of hospital admissions and longer length of stay, and even higher mortality rates [5–8]. Furthermore, a low HL level is associated with low socioeconomic status, and the impact of this is more marked in lower social status groups [6, 9, 10].

Studies that assessed the HL level of populations worldwide found a non-negligible frequency of problematic or inadequate levels in the Asian, American, and European populations [11–14]. For example, a study conducted in 8 European countries (Austria, Bulgaria, Germany, Greece, Ireland, Netherlands, Poland, Spain) found an inadequate HL level in 12% of the population and a problematic level in 35% of the population; thus, 47% of the population was considered to have a limited HL [14]. Currently, there is, to our knowledge, very little published data regarding HL level in the general population in France. However, recent data collected between 2019 and 2021 in 17 European countries for the development and validation of HLS_19_-Q12 scale seem to confirm that reported elsewhere as, when considering replies to the questions in the same manner, 44% were found to have an inadequate HL level in the French population (the mean of the 17 countries was 46%) [15].

EDs in France are easy to access since most are reachable using public transport, are open 24 hours a day, and no procedure is required before presentation [6, 16]. It is also a particular context as social vulnerability is over-represented in this population; for example, with respect to the general population, they more frequently have no or poor supplementary health insurance that leads to difficulties in accessing health care and consequently a higher frequency of ED visits [16]. In this context, during the past 20 years in France the number of consultations in ED have doubled to reach 22 million in 2019 [17, 18]. This impacts the quality of healthcare due to overwork and organizational difficulties, in a field that is already reported to be experiencing a crisis [19]. HL can be seen as a potential axis, among others, to limit the inadequate use of ED since it is associated with good healthcare use, health education and prevention [8, 20]. It is of note that individuals with limited HL seem to have greater frequency of emergency department (ED) visits [8], however the HL of patients who attend the ED has yet to be evaluated. We therefore conducted a study to evaluate the HL level of patients in a French ED.

## Material and methods

### Study design

We conducted a descriptive, cross-sectional observational, single center study in the ED of the Hôpital Lyon Sud (Hospices civils de Lyon, Lyon, France) between February 20, 2023 to March 3, 2023.

#### Population

The inclusion criteria were age ≥ 18 years and clinically stable as determined by the admission nurse; a patient is considered clinically stable when he/she is classified from 3A to 5 according to the FRench Emergency Nurses Classification in Hospitals (FRENCH) classification [21]. The exclusion criteria were severe dementia, confusion or neurological failure, psychiatric decompensation, and inability to understand French language.

#### Endpoints

The primary endpoint of this study was the HL level of the patients determined according to the score obtained using the 16-item European Health Literacy Survey Questionnaire (HLS-EU-Q16) questionnaire [22]. The secondary endpoint was the identification of sociodemographic factors associated with the HL level.

The study was approved by the ethics committee of the family medicine department of the Lyon 1 university, on December 6, 2022, and the present report follows the STROBE statement [23].

### Data collection

Data collection was performed immediately after the inclusion of the patients. After written informed consent was obtained, the study questionnaire was administered by a physician in consultation boxes or in waiting rooms when it was possible (between 9am and 4pm), given to the patient to fill-out by him/herself. To take into consideration the vulnerability of patients, and the stressful context of ED, the physician asked the questions once the patient was calm and felt able to answer the questions. For the patients with hearing loss or impaired vision the physician had to speak louder and/or ensure that hearing aids were in place, that the patient had put his/her glasses on if needed, etc. The layout was designed to make it easier to read: written in bold text,, and each reply was in a different color (red for very difficult, orange for difficult, yellow for easy and green for very easy).

The first part of the study questionnaire collected sociodemographic data which was followed by the HLS-EU-Q16. After anonymization, all questionnaires and consent forms were stored in a secure server.

### Sociodemographic characteristics

Sociodemographic variables collected were: age (categorized from 18-25 to >75 in 10-year intervals as used in the study reporting the development and validation of the HLS_19_-Q12 study [15]), sex (male or female), country of birth, native language), level of education (less than middle school, middle school, high school, more than high school), profession, perceived health status (very good, good, bad, very bad), internet access (yes or no), living environment (rural or urban), isolated (yes or no). These sociodemographic factors were chosen because they had already been reported to be associated with HL [6, 7, 12, 14, 24–27]. We added whether they were referred to the ED by a physician (either during a consultation or by telephone, including via the emergency telephone number for medical help; yes or no) could potentially been linked with HL level.

### Questionnaire

The level of HL was assessed using the French version of the HLS-EU-Q16 questionnaire, validated in 2018. It contains 16 questions with 4 possible answers: very difficult, difficult, easy, or very easy; 1 point is given for replies “easy” or “very easy”, and 0 points for “difficult” or “very difficult”. The total score ranges from 0 to 16; a HL level can be classified as inadequate or level 1 (from 0 to 8 points), problematic or level 2 (from 9 to 12 points), and adequate or level 3 (from 13 to 16 points) [22]. This multidimensional questionnaire was developed from the HLS-EU-Q47 questionnaire [2].

### Statistical analysis

Comparisons were made using the Kruskal-Wallis rank sum test for continuous variables, and the Fisher's exact test or Pearson's Chi-squared test for categorical covariates. All analyses were performed using Excel (version 15.0.5545.1000; Microsoft, Redmond, WA, US) and R (version 4.2.3; The R Foundation or Statistical Computing, Vienna, Austria) with the package ordinal.

Associations were explored using a multivariate proportional odds model using the level of literacy as the response variable, and independent covariates as predictors, which were: age (considered as a continuous variable), sex (male or female), perceived health status treated as a numerical variable (ordered scale {1, 2, 3, 4}, 1 being the worst), levels of education with a sequential effect (less than middle school, middle school, high school, more than high school), living environment (rural or urban), internet access (yes or no), and being isolated (yes or no). We chose to put as independent covariates the factors who were significant in univariate analysis and the most frequent studied in previous studies. The magnitude of association is given as odds ratio (OR) and 95% confidence interval [95% CI] for each predictor studied. The OR measures the association between the HL level and each independent predictor; for OR<1, the increase of the predictor leads to the proportional decrease of the HL level, while for OR>1, the increase of the predictor leads to the proportional increase of the HL level.

The alpha risk was set at 0.05, without correction for multiple testing. No imputation was conducted to address missing data; for modelling purposes, only the subjects for whom the HL level and covariates were available were included in the model.

## Results

### Patient characteristics

**Fig. 1:**
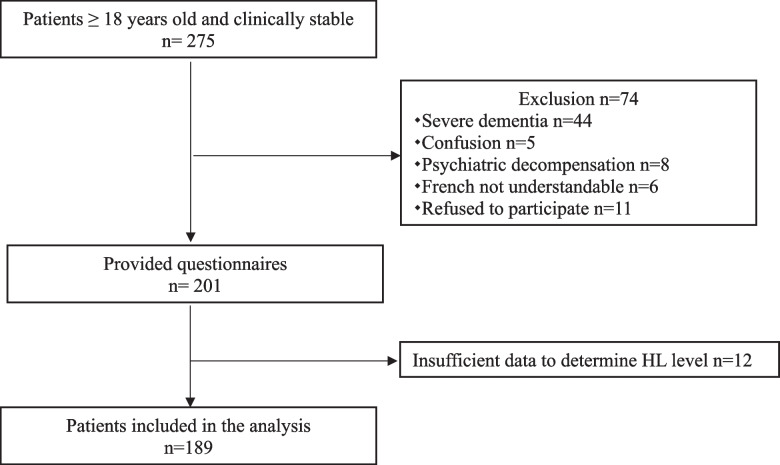
Flowchart

Among the 275 patients who met the inclusion criteria, 74 were excluded from the study; a further 12 patients were excluded from the analysis due to insufficient data to determine HL level. Thus, 189 patients were included for analysis (Fig. 1). The mean (± standard deviation) age of the patients was 60 (± 21) years. A total of 150 (80%) patients were born in metropolitan France, and French was the native language of 161 (86%) patients. The majority of the patients had obtained a diploma (middle school, high school, more than high school), and were most frequently manual workers or artisans (106, 57%) followed by office employees or intermediate professions (42, 23%); perceived health was reported to be good in 92 (49%) patients and bad in 60 (32%) patients, and 121 (64%) patients were referred to the ED by a physician. Most of the patients were not living alone (165, 88%) and the majority had an access to the internet (152, 82%; Table 1).

**Table 1:** Data description, by literacy level

			**Literacy level**^a^			
**Variable**	**N**	**Overall**, *N* = **189**^**1**^	**1**, *N* = 18	**2**, *N* = 71	**3**, *N* = 100	***p*-value**
**Age – years, Mean (SD)**	188	60 (21)	74 (21)	63 (22)	55 (20)	**<0.001**^**2**^
Unknown		1	0	1	0	
**Age – years (cat.), n (%)**	188					**0.010**^**3**^
[18,25]		13 (7)	0 (0)	5 (7)	8 (8)	
[26,35]		20 (11)	1 (6)	7 (10)	12 (12)	
[36,45]		19 (10)	2 (11)	5 (7)	12 (12)	
[46,55]		28 (15)	2 (11)	11 (16)	15 (15)	
[56,65]		23 (12)	0 (0)	5 (7)	18 (18)	
[66,75]		28 (15)	1 (6)	9 (13)	18 (18)	
[>75]		57 (30)	12 (67)	28 (40)	17 (17)	
Unknown		1	0	1	0	
**Sex, n (%)**	189					0.46^4^
Female		95 (50)	10 (56)	39 (55)	46 (46)	
Male		94 (50)	8 (44)	32 (45)	54 (54)	
**Native country, n (%)**	188					0.34^3^
France (metropolitan)		150 (80)	15 (83)	61 (86)	74 (75)	
Maghreb		18 (10)	1 (6)	3 (4)	14 (14)	
Sub-Saharan Africa		8 (4)	0 (0)	3 (4)	5 (5)	
Other^b^		12 (6)	2 (11)	4 (6)	6 (6)	
Unknown		1	0	0	1	
**Native language, n (%)**	188					0.47^3^
French		161 (86)	16 (89)	62 (89)	83 (83)	
Other^c^		16 (8)	2 (11)	6 (9)	8 (8)	
Arab		11 (6)	0 (0)	2 (3)	9 (9)	
Unknown		1	0	1	0	
**Level of education, n (%)**	189					0.24^3^
Less than middle school		30 (16)	4 (22)	15 (21)	11 (11)	
Middle school		37 (20)	6 (33)	13 (18)	18 (18)	
High school		53 (28)	5 (28)	22 (31)	26 (26)	
More than high school		69 (36)	3 (17)	21 (30)	45 (45)	
**Professional activity, n (%)**	185					0.70^3^
Manual worker, artisan		106 (57)	13 (72)	39 (57)	54 (55)	
Office employees, intermediate profession		42 (23)	5 (28)	15 (22)	22 (22)	
Higher managerial and professional positions		25 (14)	0 (0)	10 (14)	15 (15)	
Student		7 (4)	0 (0)	2 (3)	5 (5)	
Without		5 (3)	0 (0)	3 (4)	2 (2)	
Unknown		4	0	2	2	
**Perceived health status, n (%)**	189					**0.007**^**3**^
Very bad		6 (3)	0 (0)	5 (7)	1 (1)	
Bad		60 (32)	8 (44)	27 (38)	25 (25)	
Good		92 (49)	8 (44)	35 (49)	49 (49)	
Very good		31 (16)	2 (11)	4 (6)	25 (25)	
**Living environment, n (%)**	188					0.63^4^
Rural		61 (32)	5 (29)	26 (37)	30 (30)	
Urban		127 (68)	12 (71)	45 (63)	70 (70)	
Unknown		1	1	0	0	
**Isolated, n (%)**	188					0.77^3^
No		165 (88)	15 (83)	62 (89)	88 (88)	
Yes		23 (12)	3 (17)	8 (11)	12 (12)	
Unknown		1	0	1	0	
**Internet access, n (%)**	186					**0.003**^**3**^
No		34 (18)	6 (33)	19 (28)	9 (9)	
Yes		152 (82)	12 (67)	50 (72)	90 (91)	
Unknown		3	0	2	1	
**Referred to the ED by a physician, n (%)**	188					0.070^4^
No		67 (36)	8 (44)	18 (25)	41 (41)	
Yes		121 (64)	10 (56)	53 (75)	58 (59)	
Unknown		1	0	0	1	

^1^Mean (standard deviation, SD); n (%)

^2^Kruskal-Wallis rank sum test

^3^Fisher's Exact Test for Count Data with simulated *p*-value (based on 1000 replicates)

^4^Pearson's Chi-squared test

^a^1 : Inadequate 2 : Problematic 3 : Adequate

^b^Italy, Spain, Portugal, Turkey, Martinique, and Mauritius

^c^Italian, Spanish, Portuguese, Turkish, Kikongo, Baoulé, Bettie

### HL level

**Fig. 2:**
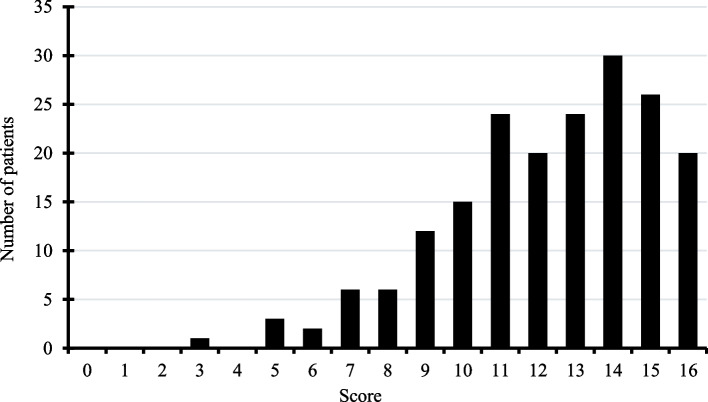
Distribution of HLS-EU-Q16 scores

Overall, 10% (95% CI [3%; 17%]) of the patients had an inadequate HL, 38% (95% CI [31%; 45%]) had a problematic HL, and 53% (95% CI [46%; 61%] had an adequate HL (Fig. 2).

### Bivariate analysis on sociodemographic factors

The statistical analysis exploring each sociodemographic factor is shown in Table 1. In bivariate analysis, three factors influenced significantly the HL level, age (*p*<0.001), a bad perceived health status (*p*<0.007), and internet access (*p*<0.003).

### Multivariate analysis

In multivariate analysis, age and perceived health status remained independent predictors of the HL level; OR =0.82 (95% CI [0.69; 0.97]; *p*=0.026) for a 10-year increase in age, and OR =1.84 (95% CI [1.22; 2.82; *p*=0.004]) for a one-category increase in perceived health status. Internet access was not significantly associated with HL level (Table 2).

**Table 2:** Final proportional odds model explaining the literacy level

**Variable**	**Effects**	
	**OR [95% CI]**^a^	***p*-value**
Age (+10 years)	0.82 [0.69; 0.97]	**0.026**
Sex		
Female	—	
Male	1.30 [0.70; 2.42]	0.41
Perceived health status (+1)	1.84 [1.22; 2.82]	**0.004**
Level of education		
Less than middle school	1.51 [0.57; 4.01]	0.41
Middle school	0.67 [0.26; 1.70]	0.41
High school	1.59 [0.72; 3.52]	0.25
More than high school	1.11 [0.20; 9.04]	0.91
Living environment		
Rural	—	
Urban	0.96 [0.50; 1.82]	0.90
Internet availability		
No	—	
Yes	1.97 [0.83; 4.77]	0.13
Isolated		
No	—	
Yes	0.99 [0.40; 2.52]	0.99

^a^*OR* Odds Ratio, *CI* Confidence Interval

Multivariate analysis were performed on 185 patients due to missing data on influencing factors

## Discussion

The present study found that nearly half of the patients had an inadequate or problematic HL level. This proportion of patients with limited HL was similar to that reported in the study conducted in the general population of European countries (47%) that used a similar type of questionnaire [14]; it is also similar to that reported in the HLS_19_-Q12 study, both in the general population of France (44%) and more generally in European countries (46%) [15]. This suggests that the population attending an ED in France is not characterized by a particularly low HL.

Several sociodemographic factors that were described as associated with HL in previous studies were assessed herein [6, 7, 12, 14, 24–27]. It has been shown that the elderly, individuals with disabilities, those with lower socioeconomic status, ethnic minorities, and those with limited education had a lower HL level [28]. In the present study, only age and perceived health status were independent predictors of HL level; younger individuals were more likely to have an adequate HL, while those with bad perceived health status were more likely to have limited HL. The association of age with HL found herein is consistent with previous studies [14, 24–26], the influence of age on HL is the most constant finding in the literature. A positive association between perceived health status and HL is also frequently reported [14, 25]. The elderly and individuals with bad perceived health status are those who most use the healthcare system [29], underlining the importance of considering their HL level. Sudor and Schillinger have investigated this and identified several solutions including methods to improve oral communication, the use of supports such as videos, pictures, *etc.*, and the benefit of providing written information [30]. In France, Dr Melanie Sustersic and her team worked on written information that can be provided to the patients, by drafting clear information sheets on frequently encountered medical subjects and chronic diseases, which could be a way to provide easy information that can be read several times and at home [31].

Internet access herein was significantly associated with HL in the bivariate analysis but was not found to be an independent predictor of HL. Internet access and its influence is not often studied but seems to be related with adequate HL; for instance, Protheroe *et al*. reported a significant association between limited HL and the absence of internet access in England [26], and a recent meta-analysis of modifiable predictors of HL in working-age-adults found a positive association between adequate HL and a high frequency of internet use as well as using internet as a source of information [27]. This could be explained by the access to information that the internet makes possibly, but also the digital landscape that characterizes the modern world; it is also of note that eHealth literacy is now a dedicated sub-domain of HL [32, 33].

In the present study, education level or the socioeconomic status was not found to be associated with HL, although these are the most consistent findings in the literature [12, 14, 25, 26]. This is probably due to the overestimation of the educational level in the category “high school’ as this includes (in France) both professional/vocational training as well as more academic (class-room) education. Moreover, there was no influence of the sex herein, which is inconsistent with several studies that found a better HL level in women [6, 7, 12, 14] although others did not find this [24, 26]. However, being isolated, as well as living in rural or urban environments, were also not predictive factors, which is consistent with the studies that have explored these aspects [12, 26].

Interestingly, the majority of patients in the present study had received medical advice before going to the ED, but over a third did not. Although, there was no association with HL level in the present study, it could be interesting to conduct studies to further explore this; for instance, whether this is related specifically to difficulties to make an appointment, to reach a physician, or perhaps evaluate whether there is a real emergency or not.

It could be hypothesized that taking into consideration the HL level of the population could have an impact on the patient flow to ED by reducing the number of patients who can be treated in another structure, reduce the occurrence of chronic disease decompensation, avoid iatrogenic incidents due to errors or misunderstanding, and increase prevention and better health. Few studies assessed the relationship between HL and ED flows but found divergent results, such as those reported by Rasu et al. who found a significant association between frequency of ED visits and inadequate or limited HL in the US [8], but Vandenbosch et al. did not find any relationship between HL level and ED visits in Belgium [6].

## Limitations

This study has several limitations. The first is the small size of the sample giving a weak power and the short assessment period, but it is of note that there were relatively few missing data. It could also be interesting to compare HL level in patients attending the ED with the general population of France, as well as between ED in France to investigate whether there is any difference between hospitals (rural, urban, localization, etc.). The second is its single-center nature that could lead to selection bias. Moreover, some limitations are related to the HLS-EU-Q16 questionnaire that leads to response bias as some questions can be ambiguous, complex, or mind-setting. For example, during administration by a physician some questions seemed easy for the patients, but others very difficult to understand, and some patients could not decide between the answer ‘easy’ and ‘difficult’; there is also the possibility of an influence by the way the questions were asked and the stressful and particular context of ED. Self-reporting is also to be taken into account as potential non-negligible bias leading to a possibility of overestimation of HL level. However, despite its limitations, the HLS-EU-Q16 is a validated and functional psychometric questionnaire [22]. Furthermore, it is the first to provide a first description of HL in the specific context of ED and contribute to the recognition of HL among healthcare professionals in the ED and to take into account HL level.

## Conclusion

The HL level of the patients in the ED studied herein was similar to that found in the population of France and other European countries and was influenced by age and perceived health status, which are both associated with care needs. It may be therefore interesting to explore in future studies how taking into consideration HL in the general population may lead to a better self-efficacy in care and optimize the use of the healthcare system.

## Data Availability

The datasets used and/or analyzed in the present study are available upon reasonable request to the corresponding author. Data transfer must be approved by the French data protection agency (*Commission nationale de l’informatique et des libertés*, CNIL).

## Abbreviations

HL: Health literacy
HLS-EU-Q16: 16-item European Health Literacy Survey Questionnaire
ED: Emergency department
HLS-EU-Q47: 47-item European Health Literacy Survey Questionnaire
OR: Odds Ratio
CI: Confidence interval
STROBE: Strengthening the Reporting of Observational Studies in Epidemiology
FRENCH Classification: FRench Emergency Nurses Classification in Hospitals
SD: Standard deviation
